# Comprehensive bibliometric analysis of sirtuins: Focus on sirt1 and kidney disease

**DOI:** 10.3389/fphar.2022.966786

**Published:** 2022-08-16

**Authors:** Tongtong Liu, Shujuan Mu, Liping Yang, Huimin Mao, Fang Ma, Yuyang Wang, Yongli Zhan

**Affiliations:** ^1^ Guang’anmen Hospital, China Academy of Chinese Medical Sciences, Beijing, China; ^2^ South District of Guang’anmen Hospital, China Academy of Chinese Medical Sciences, Beijing, China

**Keywords:** bibliometric analysis, SIRT1, kidney disease, NAD, oxidative stress, autophagy

## Abstract

Sirtuins, as regulators of metabolism and energy, have been found to play an important role in health and disease. Sirt1, the most widely studied member of the sirtuin family, can ameliorate oxidative stress, immune inflammation, autophagy, and mitochondrial homeostasis by deacetylating regulatory histone and nonhistone proteins. Notably, sirt1 has gradually gained attention in kidney disease research. Therefore, an evaluation of the overall distribution of publications concerning sirt1 based on bibliometric analysis methods to understand the thematic evolution and emerging research trends is necessary to discover topics with potential implications for kidney disease research. We conducted a bibliometric analysis of publications derived from the Web of Science Core Collection and found that publications concerning sirt1 have grown dramatically over the past 2 decades, especially in the past 5 years. Among these, the proportion of publications regarding kidney diseases have increased annually. China and the United States are major contributors to the study of sirt1, and Japanese researchers have made important contributions to the study of sirt1 in kidney disease. Obesity, and Alzheimer’s disease are hotspots diseases for the study of sirt1, while diabetic nephropathy is regarded as a research hotspot in the study of sirt1 in kidney disease. NAD^+^, oxidative stress, and p53 are the focus of the sirt1 research field. Autophagy and NLRP3 inflammasome are emerging research trends have gradually attracted the interest of scholars in sirt1, as well as in kidney disease. Notably, we also identified several potential research topics that may link sirt1 and kidney disease, which require further study, including immune function, metabolic reprogramming, and fecal microbiota.

## Introduction

**FIGURE 1:**
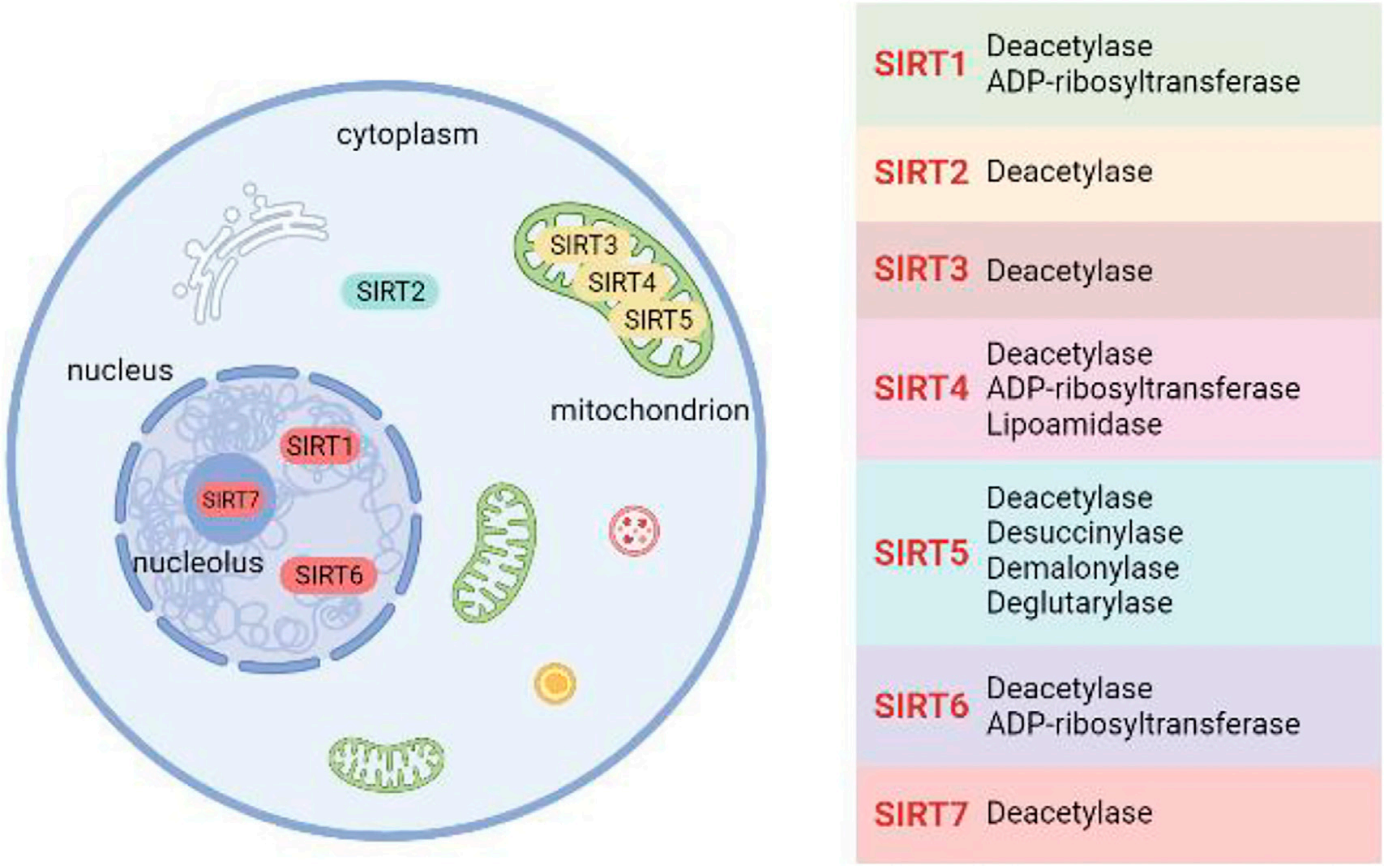
The role of sirtuins in kidney disease.

Sirtuins belong to a highly evolutionarily conserved histone deacetylase (HDACs) family that is dependent on nicotinamide adenine dinucleotide (NAD^+^) (Wang and Lin, 2021). Silent information regulator 2 (Sir2) was originally identified in yeast and named in 1987 (Ivy et al., 1986). Subsequently, Sir2 was found to be involved in regulating the replicative lifespan of yeast, increasing the dose of Sir2 increased yeast lifespan by 30% (Kaeberlein et al., 1999). Sirtuins are homologous mammalian enzymes of Sir2 and consist of seven isoforms (sirt1-sirt7). The intracellular localization of Sirtuins is closely related to its function. Sirt1, sirt6, and sirt7 are mainly located in the nucleus, and primarily responsible for transcriptional regulation, repair of DNA and regulation of the cell cycle. Sirt3-sirt5 exist in mitochondria, and plays an important role in regulating cell energy metabolism. Sirt2 is the only directly available family member in the cytoplasm, and mainly functions in mitosis (Zhao et al., 2020). All members of the Sirtuins family have deacetylation properties, sirt1, sirt4, and sirt6 have been found to possess ADP-ribosyltransferase activity, sirt5 and sirt7 have also been reported to be a desuccinylase (Srivastava et al., 2020b; Gupta et al., 2022). Notably, sirt5 also has two unique properties of demalonylation and deglutarylation (Kumar and Lombard, 2018). Sirt4 has been reported to possess unique lipoamidase activity (Mathias et al., 2014) (Figure 1). Sirtuins, as a regulator of metabolism and energy, are closely associated with many aging-related diseases, including cardiovascular diseases (Soni et al., 2021), cancer (Aventaggiato et al., 2021), and neurodegenerative disorders (Leite et al., 2022). Accumulating evidence suggests that sirtuins play important roles in transcriptional regulation, cell survival, cell stress resistance, DNA damage and repair, and cell cycle regulation (Haigis and Sinclair, 2010). Interestingly, sirtuins were found to be central players in health improvement associated with calorie restriction and exercise training. In addition, some natural compounds (such as resveratrol, astragaloside IV, and honokiol) and small molecules (such as SRT1720, SRT1460, and SRT3025) have also been found to ameliorate diseases by activating sirtuins, which has made scholars realize the importance of sirtuins as potential pharmacological targets for many diseases (Dai et al., 2018).

Sirt1 is phylogenetically similar to yeast Sir2 and is the most extensively studied member of the sirtuin family. Sirt1 can regulate energy metabolism, immune inflammation, oxidative stress, mitochondrial homeostasis, autophagy, and apoptosis, and in turn ameliorates disease progression and aging by deacetylating regulatory histones and nonhistone proteins, such as peroxisome proliferator-activated receptor alpha (PPARα), PPAR gamma coactivator-1alpha (PGC-1α), and fork-head box protein (FOXO) (Zhao et al., 2020). The kidney is one of the major organs that are prone to aging-related diseases, especially in diabetes mellitus conditions. Multiple stresses lead to accelerated renal aging, including accumulation of advanced glycation end products, inflammation, autophagic damage, and oxidative stress (Guo et al., 2020). The study of sirt1 in kidney disease, especially in diabetic nephropathy (DN), has gradually gained the attention of researchers. On the one hand, sirt1 can improve renal resident cell injury and apoptosis by directly regulating oxidative stress, mitochondrial homeostasis, and autophagy. On the other hand, sirt1 can modulate ectopic lipid accumulation in the kidney, ameliorate fibrosis, and prevent the progression of renal disease. In addition, sirt1 has also been found to ameliorate vascular endothelial injury and reduce complications of kidney disease, thereby improving the vulnerability of the kidney to aging (Morigi et al., 2018). Studies have found that the knockout of renal sirt1 can lead to the aggravation of inflammation, proteinuria, and fibrosis (He et al., 2010; Liu et al., 2014), and these phenotypes of renal injury may be significantly ameliorated by using resveratrol (RSV) (Kitada et al., 2011), NMN (Yasuda et al., 2021), or SRT1720 (Funk et al., 2010) to activate sirt1. Thus, sirt1 plays an irreplaceable role in the initiation and progression of kidney diseases.

Bibliometric analysis is an effective way to assess overall trends in a field. It not only helps researchers and clinicians understand core countries, institutions, and authors of a given research area and the most influential nodal publications, but also identifies thematic changes, emerging research trends, and research gaps in this area (Sabe et al., 2022). In recent years, there has been an explosion of research on the role of sirtuins in health and disease. However, studies evaluating the overall trends of sirtuins are scarce. To our knowledge, only one bibliometric analysis has evaluated sirt6 (Lu et al., 2018). Therefore, in this study, we evaluated the overall distribution of studies on sirtuins over the past 2 decades using the bibliometric analysis method, and with a particular focus on sirt1 and kidney disease. Through an analysis of the main research areas and emerging research trends in the field of sirt1 research, we hope to shed new light and ideas on the study of sirt1 in kidney disease.

## Methods

### Search strategy

The Web of Science Core Collection (WoSCC), the most comprehensive database for bibliometric analysis, was used to conduct a comprehensive search of publications related to sirtuins. We used topic subject (TS) as our search strategy, and the retrieval formula was set as TS = (“sirt” OR “sirtuin” OR “sirtuins” OR “SIRT1” OR “sirtuin1” OR “SIRT2” OR “sirtuin2” OR “SIRT3” OR “sirtuin3” OR “SIRT4” OR “sirtuin4” OR “SIRT5” OR “sirtuin5” OR “SIRT6” OR “sirtuin6” OR “SIRT7” OR “sirtuin7”). Publication type was restricted to “article” and “review”. In addition, we performed additional analyses of sirtuin family members (sirt1-sirt7) separately, with a focus on the analysis of sirt1 and sirt1 in kidney disease. The retrieval formula is shown in Supplementary Table S1. The above process was completed in 1 day on 25 April 2022, to avoid bias from data updates.

### Data analysis

Citespace (version 6.1.R1), VOSviewer (version 1.6.16), Bibliometrix 4.1.0 package (https://www.bibliometrix.org), and the Arrowsmith project (http://arrowsmith.psych.uic.edu) were used to analyze the collected data, including the overall distribution of publications, leading countries and institutions, core journals, active authors, co-citation references, and keyword analysis. The Journal Citation Reports (JCR) and Hirsch index (H-index) have also been used to assist in assessing the academic impact of journals and authors.

Citespace (Chen, 2004) was used to perform cluster analysis and burst analysis of co-references and keywords to understand the main research areas and emerging research trends of sirt1, and automated labelling for cluster interpretation. We defined modularity Q > 0.3 and mean silhouette >0.5 as indicators that the clustering results were sufficiently stable and reliable. Bibliometrix, based on the R project (Aria and Cuccurullo, 2017), was used to construct the thematic evolution of keywords, and the time cutting points were set as 2018 and 2020, respectively (recent five and 3 years). Next, VOSviewer (van Eck and Waltman, 2010) was used to extract and visualize the keywords for sirt1 in kidney disease research. Finally, the Arrowsmith project (Smalheiser et al., 2009) was used to construct an association between sirt1 and kidney disease. The retrieval formula of A-query (sirt1) was set as [sirt1 (MeSH Terms)] OR [sirtuin1 (MeSH Terms]), and the retrieval formula of C-query (kidney disease) was set as [kidney disease (MeSH Terms)] OR [renal disease (MeSH Terms)] OR [nephropathy (MeSH Terms)]. Using the keywords obtained from the Arrowsmith Project as the prediction group and the keywords extracted from VOSviewer was used as the confirmation group, and a Venn diagram of the two groups was drawn to obtain the potential links between sirt1 research and kidney disease. String (https://cn.string-db.org) was used to construct protein-protein interaction (PPI) networks for the target proteins.

## Results

### Overall distribution and publication trends of sirtuins

**FIGURE 2:**
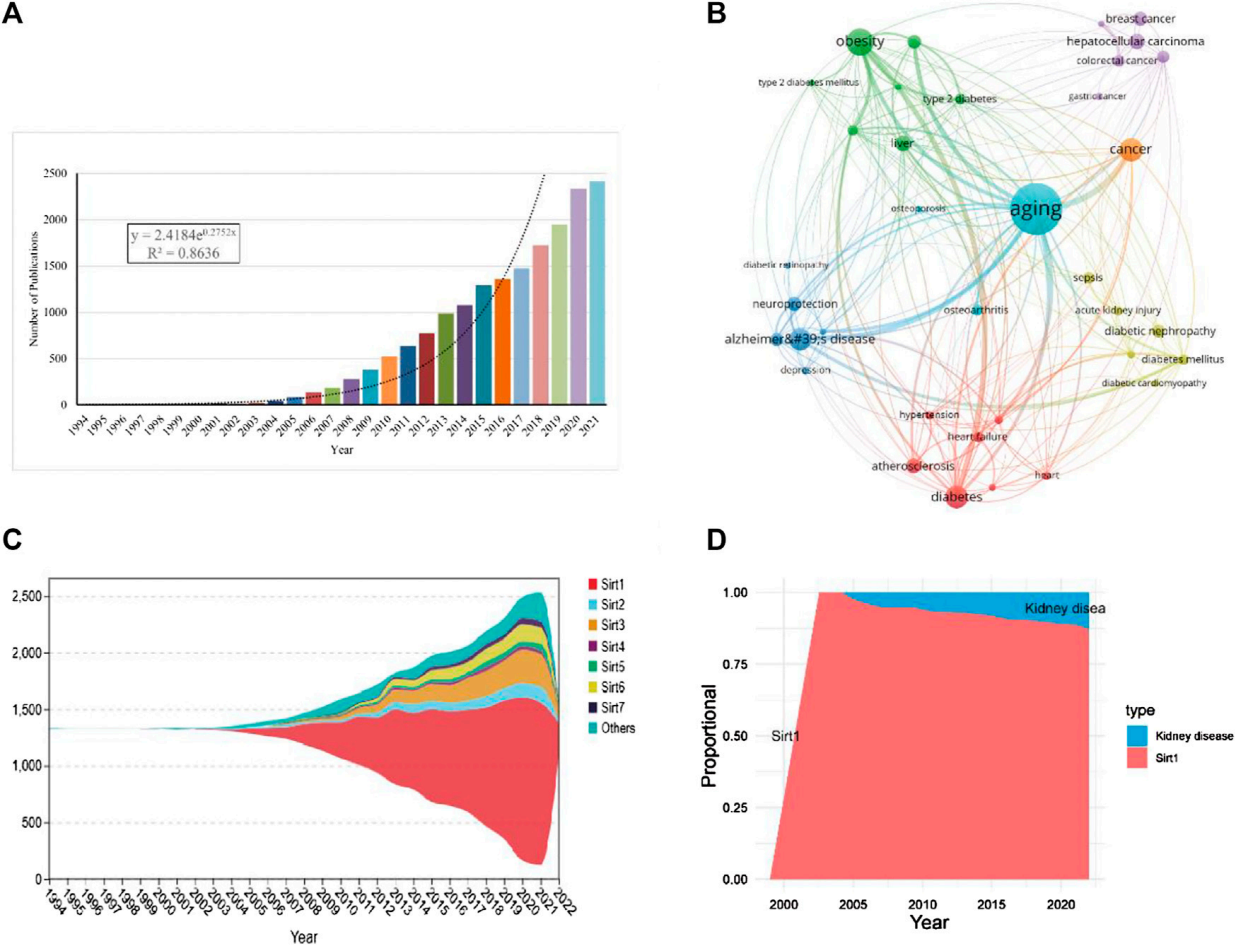
Overall distribution of sirtuins study. **(A)**. Global annual production trends in studies on sirtuins. **(B)**. Key diseases in sirtuins related research. **(C)**. Stream plots of annual production trends for the sirtuins family. **(D)**. The annual proportion of kidney disease in studies on sirt1.

A total of 18225 publications from 1994 to 2022 were retrieved from the WoSCC. Through curve fitting analysis, we found that the publications about sirtuins showed a sharp growth trend with an annual growth rate of 13.10% in the last 5 years (*R* ^2^ = 0.8386, Figure 2A). From 1994 to 2004, studies on sirtuins were in their infancy, with no more than 50 articles published annually. Since 2005, the number of publications on sirtuins has increased dramatically at an average rate of 100 per year. Even from 2013, the growth rate of publications on sirtuins reached an average of 200 publications per year, illustrating that research on sirtuins has gradually emerged in numerous fields.

Through co-occurrence analysis of the keywords of the retrieved publications, it was found that the diseases related to sirtuins research are mainly concentrated in aging, obesity, neurodegenerative diseases, cancer, and cardiovascular diseases (Figure 2B; Table 1). Publications about sirt1, growing rapidly (*R* ^2^ = 0.9733), were the most studied (*n* = 11265, 61.81%) and had the highest h-index (250) (Figure 2C; Table 2). There were 744 articles focusing on kidney disease, and the proportion of research investigating kidney disease and sirt1 increased annually (Figure 2D), suggesting that an increasing number of scholars have focused on the potential role of sirt1 in kidney disease. Next, we present an in-depth analysis of publications on sirt1, with a particular focus on the field of kidney disease.

**TABLE 1:** Top ten diseases most significantly associated with sirtuins in publications.

Rank	Disease	Occurrences
1	Aging	822
2	Obesity	340
3	Cancer	273
4	Diabetes	266
5	Alzheimer’s disease	259
6	Liver	163
7	Hepatocellular Carcinoma	161
8	Atherosclerosis	154
9	Neuroprotection	144
10	Breast cancer	142

**TABLE 2:** Overall distribution of publications in sirtuin family.

Sirtuins	Enzyme activity	Number of publications	Total times cited	Average citation frequency	Growth factor (*R* ^2^,2011–2021)	Annual growth rate (2017–2021)	H (%)-index
Sirtuins		18225	710588	38.98974	0.9852	13.10	304
Sirt1	Deacetylase, ADP-ribosyltransferase	11265 (61.81%)	454744	40.36787	0.9733	12.29	250
Sirt2	Deacetylase	1061 (5.82%)	42078	39.65881	0.9074	12.80	97
Sirt3	Deacetylase	2049 (11.24%)	80064	39.07467	0.9528	10.06	126
Sirt4	Deacetylase, ADP-ribosyltransferase, Lipoamidase	289 (1.58%)	16779	58.05882	0.6897	10.23	54
Sirt5	Deacetylase, desuccinylase, demalonylase, deglutarylase	390 (2.14%)	19871	50.95128	0.8766	24.56	64
Sirt6	Deacetylase, ADP-ribosyltransferase	1039 (5.70%)	42127	40.54572	0.8867	11.27	98
Sirt7	Deacetylase	363 (1.99%)	19500	53.71901	0.8454	8.68	64

### Country, institution, and author analysis

**FIGURE 3:**
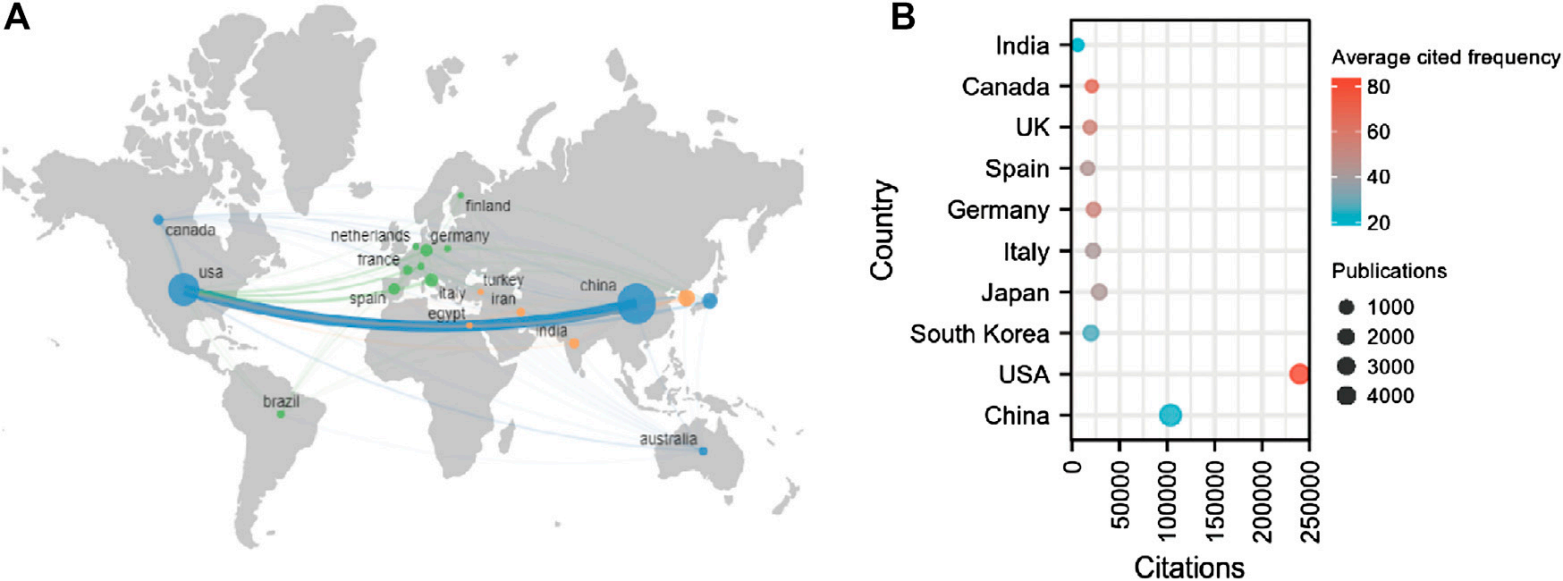
National contributions of the sirt1 study. **(A)** Global geographic distribution and collaboration regarding the study of sirt1. **(B)** The top ten highest productive countries for the study of sirt1.

In total, 6941 institutions from 92 countries contributed to publications related to sirt1. The United States is a central player in international collaboration and has partnered with many countries, most notably with China (Figure 3A).75.68% of publications were represented by the top ten countries with the largest number of publications (Table 3). China and the United States are far ahead of other countries in terms of the number of publications and total citations. In terms of average citation frequency, the United States (average cited 83.60 times) continues to rank first, followed by Canada (average cited 67.18 times) and the United Kingdom (average cited 53.94 times) (Figure 3B). Furthermore, seven of the ten most cited institutions worldwide are from the United States, which explains why the United States is far ahead of the world in terms of the total and average number of citations. In the field of kidney disease, China and the United States are still far ahead in terms of the number of publications and citations. However, Japan ranked first in terms of average citation frequency, followed by Italy and the United States (Supplemental Table S2). Kanazawa Medical University (cited 1799 times) and Shiga University of Medical Science (cited 1495 times) from Japan and Fudan University (cited 958 times) from China are the most influential institutions in the field of sirt1 in kidney diseases.

**TABLE 3:** The top 10 highest productive countries for the study of sirt1.

Rank	Country	Number of publications	Total times cited	Average citation frequency	H-index
1	China	4904 (43.53%)	103654	21.14	106
2	United States	2870 (25.48%)	239938	83.60	222
3	South Korea	762 (6.76%)	19723	25.88	66
4	Japan	673 (5.97%)	28504	42.35	86
5	Italy	524 (4.65%)	22025	42.03	74
6	Germany	424 (3.76%)	22516	53.10	75
7	Spain	377 (3.35%)	16498	43.76	65
8	United Kingdom	345 (3.06%)	18608	53.94	70
9	Canada	309 (2.74%)	20758	67.18	70
10	India	309 (2.74%)	5788	18.73	40

A total of 39208 authors contributed to the study of sirt1. Frye RA from the University of Pittsburgh was the first scholar to publish publications on sirt1 in 1999 (Frye, 1999). Sinclair DA (cited 20460 times) from Harvard Medical School and Guarente L (cited 18758 times) from Massachusetts Institute of Technology (MIT), with the highest number of citations, were the most influential authors in terms of sirt1 research (Table 4). In the field of kidney disease, 3789 authors have been involved in the study of sirt1. Koya D from Kanazawa Medical University (cited 1799 times) and Kume S from Shiga University of Medical Science (cited 1446 times), as the most cited authors, were the most influential scholars (Supplemental Table S3). Koya D and Kume S were also the first to publish that sirt1 plays a key role in kidney disease.

**TABLE 4:** The top five most cited authors for the study of sirt1.

Rank	Author	Institution	Country	Number of publications	Total times cited	Average citation frequency	H-index
1	Sinclair DA	Harvard Medical School	United States	66	20460	310.00	48
2	Guarente L	Massachusetts Institute of Technology	United States	63	18758	297.75	52
3	Auwerx J	Ecole Polytechnique Fédérale de Lausanne	Switzerland	50	16415	328.30	39
4	Puigserver P	Harvard Medical School	United States	17	12031	707.71	17
5	Mostoslavsky R	Harvard Medical School	United States	21	11734	558.76	20

### Journal analysis

**FIGURE 4:**
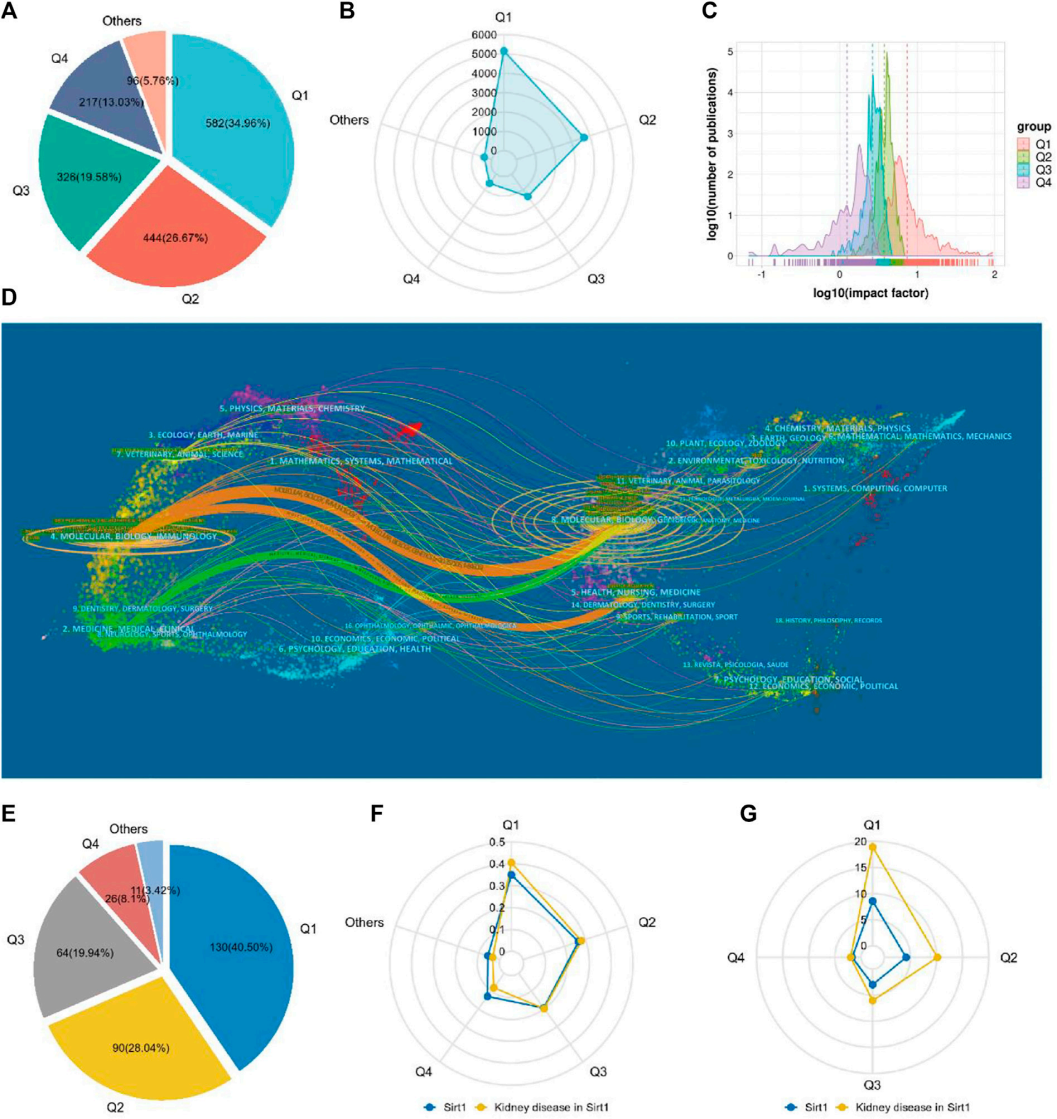
Characteristics of the core journals involved in the study of sirt1. **(A)** The quartile ranking of journals concerned with sirt1. **(B)** Radar plot of publication volume of sirt1 for journals in different quartile rankings. **(C)** Impact factor distribution of journals in different quartile rankings. **(D)** The dual-map overlay of the publications concerning sirt1. **(E)** The quartile ranking of journals concerned with sirt1 in kidney disease. **(F)** Radar plot of the proportion of different quartile rankings with respect to the number of publications of sirt1 and sirt1 in kidney disease. **(G)** Radar plot of Impact factor distribution of journals in different quartile rankings of sirt1 and sirt1 in kidney disease.

A total of 1665 journals published publications on Sirt1. According to the JCR, 582 (34.96%) journals appeared in JCR quartile 1 (Q1) and 444 (26.67%) journals were ranked in Q2 (Figure 4A), revealing the enormous academic impact of sirt1 research. Furthermore, a total of 5153 publications were published in these Q1 journals and 3690 in Q2 journals (Figure 4B), with mean impact factors of 8.55 and 4.18, respectively (Figure 4C). The Journal of Biological Chemistry, Cell, and Nature, as the journals with the highest academic impact in this field, are the three most cited journals, and eight of the ten most cited journals belong to the Q1, where Science, Nature, and Cell were the most frequently cited journals on average (Table 5). However, only three of the ten highest-volume journals appeared among the highly cited journals (Table 5). The dual-map overlay shows a total of three main citation paths in publications related to sirt1, where the publications performing citations were focused on the molecular, biology, immunology and medicine, medical, clinical field and the cited references were focused on the molecular, biology, genencs and health, nursing, medicine field (Figure 4D). In the field of kidney disease, 321 journals published relevant publications, 130 (40.5%) of which belonged to Q1, and 90 (28.04%) appeared in Q2 (Figure 4E), with mean impact factors of 18.91 and 10.18, respectively, which were better than all the publications on sirt1 (Figures 4F,G), illustrating the potential promise of sirt1 in kidney disease research. Journal of Clinical Investigation, Journal of the American Society of Nephrology (JASN), Aging Cell, and Kidney International (KI), as the most cited journals, were identified as the most influential journals (Supplemental Table S4).

**TABLE 5:** Top ten journals with the largest number of publications and the most cited related to sirt1.

Rank	Journal	Number of publications	Total times cited	Average citation frequency	Journal (cited)	Number of publications	Total times cited	Average citation frequency
1	Plos one	309	14008	45.33	Journal of biological chemistry	181	20464	113.06
2	Biochemical and Biophysical Research Communications	211	8527	40.41	Cell	30	17476	582.53
3	Scientific reports	192	5254	27.36	Nature	21	17023	810.62
4	International Journal of Molecular Sciences	189	3051	16.14	Plos one	309	14008	45.33
5	Journal of biological chemistry	181	20464	113.06	Cell metabolism	47	13207	281.00
6	Oxidative Medicine and Cellular Longevity	136	3282	24.13	PNAS	56	10756	192.07
7	Frontiers in pharmacology	130	1715	13.19	Science	13	9496	730.46
8	Aging-us	125	3363	26.90	Biochemical and biophysical research communications	211	8527	40.41
9	Molecular medicine reports	123	1926	15.66	Molecular cell	31	6574	212.06
10	Oncotarget	115	3340	29.04	Embo journal	17	5699	335.24

### Co-cited references analysis

In the top 10 most cited publications, a paper published by Lagouge et al. (2006) in Cell in 2006 was the most co-cited, with 3029 citations. This research reported that RSV, as an activator of sirt1, can promote energy and metabolic homeostasis and improve aerobic capacity. The article published in Nature by Howitz et al. (2003) was cited 2833 times and ranked second. They similarly reported that small molecules, such as RSV, can activate sirt1 and extend the lifespan of *Saccharomyces cerevisiae*. This underscores the importance of investigating sirt1 activating compounds (STACs). In addition, we analyzed the top ten most-cited publications in the last 5 years. The most cited article was written by Das et al. (2018) in 2018 and published in Cell. They reported that endothelial sirt1 deficiency is a reversible cause of vascular aging, and the NAD precursor NMN can reverse this change through sirt1. The second ranked article by citation number was published in the Journal of Neuroinflammation by Chen et al. (2018) in 2018, which showed that ω-3 polyunsaturated fatty acid supplementation can regulate HMGB1/NF-κB pathways through sirt1-mediated deacetylation to attenuate inflammatory responses. In terms of kidney disease, the most cited articles reported that RSV attenuates proteinuria, renal immunoglobulin deposition, and glomerulonephritis in lupus nephritis mice (Wang et al., 2014). In the last 5 years, the most cited articles were published by Ren et al. (2020), who reported that metformin, a classic hypoglycemic agent, produced renoprotective effects against diabetes *via* the AMPK/SIRT1-FoxO1 pathway. The second most focused article was a review published by Ralto et al.(2020), who reported that NAD^+^ homeostasis plays an important role in kidney disease and health.

**FIGURE 5:**
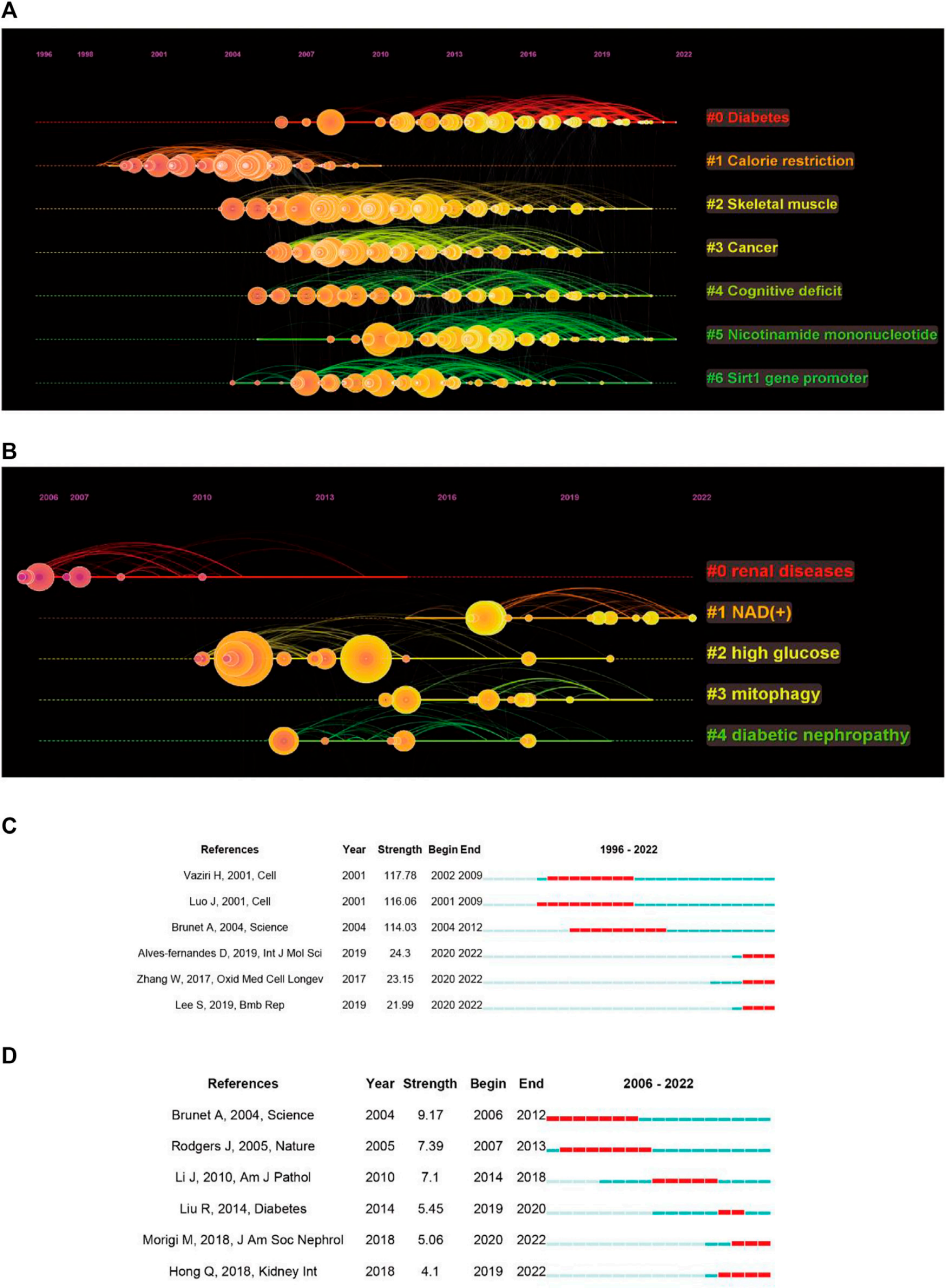
Co-cited references analysis. **(A)** Timeline plot of clustering concerning sirt1. **(B)** Timeline plot of clustering concerning sirt1 in kidney disease. **(C)** Burst analysis concerning sirt1. **(D)** Burst analysis concerning sirt1 in kidney disease.

Next, we performed a cluster analysis of the co-cited references, resulting in seven clusters with a modularity Q of 0.646 and a mean silhouette value of 0.8156 (Figure 5A). The top five clusters were enriched for diseases associated with sirt1, including diabetes, skeletal muscle, cancer, and cognitive deficit. It is important to note that nicotinamide mononucleotides (NAD) appear to be a hotspot that gains considerable interest. When focusing on kidney disease, five clusters were extracted, with a modularity Q of 0.4116 and a mean silhouette value of 0.6787 (Figure 5B). We found that NAD^+^ has also been the focus of research regarding sirt1 in kidney disease.

In addition, we performed an analysis of burst and obtained 778 burst references (Figure 5C). The reference with the strongest beginning of citation burst was published in Cell by Vaziri et al. (2001) in 2001. They described that sirt1 can deacetylate regulated p53, thus improving growth arrest or apoptosis. Similarly, the references with the second strongest beginning of citation burst were published by Luo et al. (2001) in Cell in 2001 and reported similar results. This illustrates that the regulation of deacetylation of p53 by sirt1 is a major discovery in sirt1 research. When focusing on the last 3 years, the reference with the strongest beginning of citation burst was published by Alves-fernandes D et al. and Zhang W et al., and they jointly focused on the important crosstalk role of sirt1 and oxidative stress in different diseases (Zhang et al., 2017; Alves-Fernandes and Jasiulionis, 2019). In terms of kidney disease, 49 burst references were found (Figure 5D), the publication by Brunet A et al. in Science in 2004 had the strongest beginning of citation burst, followed by Rodgers J et al., published in Nature in 2005, indicating that the regulation of FOXO and PGC-1α by sirt1 has received much attention from scholars in the field of kidney disease (Brunet et al., 2004; Rodgers et al., 2005). In the past 5 years, the publication by Liu R et al. in Diabetes in 2014 showed the strongest beginning of citation burst and found that transcription factors, such as p65 and STAT3, acetylation plays an important role in DN (Liu et al., 2014), followed by an article in JASN published by Morigi M et al. in 2018, who reviewed the central role of sirt1 in kidney health and disease (Morigi et al., 2018).

### Keyword analysis

**FIGURE 6:**
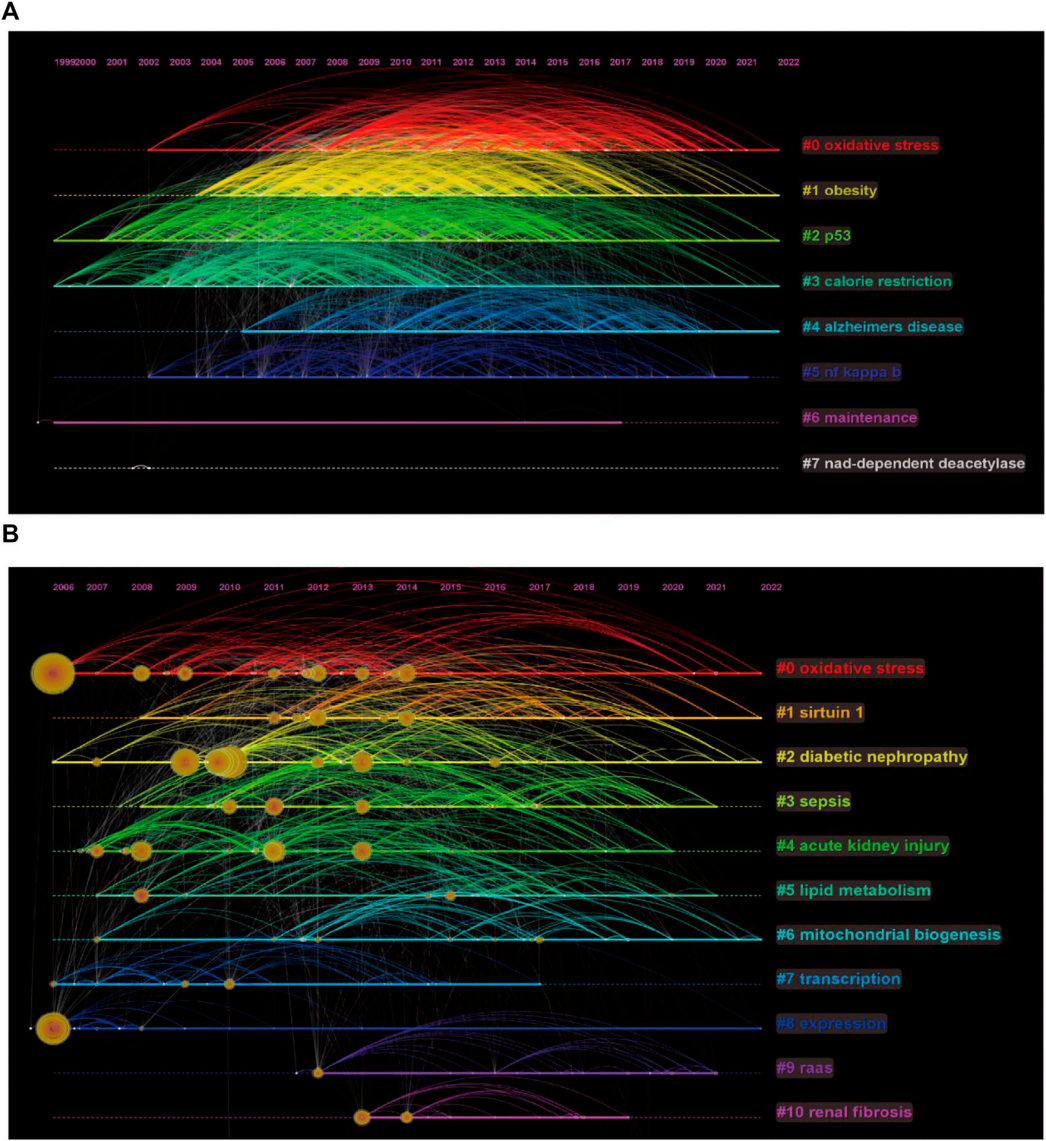
Keywords timeline of clustering. **(A)** Keywords timeline of clustering of sirt1. **(B)** Keywords timeline of clustering of sirt1 in kidney disease.

We performed clustering analysis on the extracted 24819 keywords and obtained eight clusters (Figure 6A). Obesity and Alzheimer’s disease appear to be continuing hotspots for comparative attention among scholars investigating sirt1. In addition, regulation of oxidative stress and p53 by sirt1 appears to be a hotspot of ongoing interest in mechanistic studies. In terms of kidney disease, DN is a disease of considerable interest, and oxidative stress and mitochondrial biogenesis are mechanistic studies that have received continued attention (Figure 6B).

**FIGURE 7:**
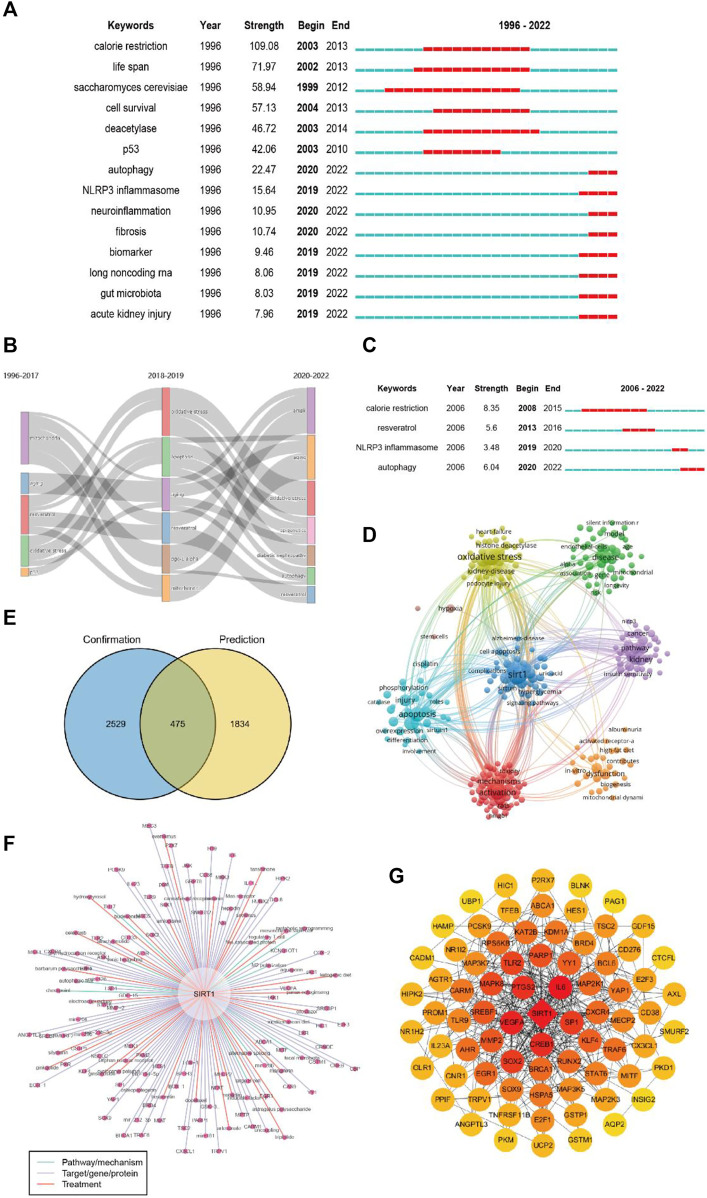
Keyword analysis. **(A)** Burst analysis concerning sirt1. **(B)** The thematic evolution of keywords. **(C)** Burst analysis concerning sirt1 in kidney disease. **(D)** Clustering of keywords related to sirt1 in kidney disease. **(E)** Venn diagram for the keywords linking sirt1 and kidney diseases. **(F)** Potential research topics linking sirt1 and kidney diseases. **(G)** PPI network for the targets related to sirt1 in kidney diseases.

A total of 331 keywords were retrieved from the burst analysis (Figure 7A). “Calorie restriction,” “life span,” and “saccharomyces cerevisiae,” as the keywords with the strongest citation bursts, indicate major areas in the study of sirt1. In the past 5 years, “autophagy,” “injury,” and “NLRP3 inflammasome” represent emerging areas in the study of sirt1. In addition, we found that the research theme of sirt1 changed over time (Figure 7B), in the last 3 years, “AMPK,” “diabetic nephropathy” and “autophagy” have been the themes of greatest interest. In terms of kidney disease (Figure 7C), 13 keywords were retrieved from burst analysis. “Calorie restriction,” “autophagy,” and “acetylation,” as the keywords with the strongest citation bursts, indicate major areas in the study of sirt1 in kidney disease, and “autophagy” and “NLRP3 inflammasome” also represent emerging areas in the past 5 years in the study of sirt1 in kidney disease.

In addition, we extracted and clustered the keywords of publications based on sirt1 studies involving kidney disease using VOSviewer. A total of 3004 keywords appeared more than five times, and these keywords formed eight clusters (Figure 7D), including sirt1-associated kidney disease (green clusters), mechanisms (purple, blue, yellow, and aquamarine clusters), and treatment options (red clusters).

Next, we explored possible connections between sirt1 and kidney disease based on the Arrowsmith project. After removing duplicates and synonyms, a total of 2309 keywords with correlations greater than 0.5 were obtained. We defined the keywords obtained from the Arrowsmith project as the “prediction” group and the keywords extracted from VOSviewer as the “confirmation” group. We constructed a Venn diagram for these two groups of keywords in the hope of discovering the keywords revealing kidney disease (Figure 7E). A total of 1834 keywords were found to serve as potential research topics linking sirt1 and kidney diseases, and the keywords with a correlation greater than 0.95 are shown in Table 6 and Figure 7F. We further constructed a PPI network for these targets and found that IL6, SP1, CREB1, VEGFA, and PTGS2 were the targets most closely related to sirt1 in kidney diseases (Figure 7G).

**TABLE 6:** Potential links between Sirt1 and kidney disease.

Rank	Correlation probabilities	Target/gene/protein	Pathway/mechanism	Treatment
1	0.99	Hepcidin, CD38, PARP1, PCSK9, CD133	Ferroptosis, immune	Hydroxytyrosol, sirolimus
2	0.98	RUNX2, LSD1, COX-2, SOX9, CXCR4, E2F1, TLR2, TSC2, BRCA1, MMP-2, osteoprotegerin, TRPV1, B7-H3, HES1, CREB, EGR1, NSCLC, TFEB, inducible factor-1, LXR, IL6, HIPK2, H19, MEK1, cannabinoid receptor, aryl hydrocarbon receptor, VEGFA, KLF4, GSK-3β, MITF, HIC1, KCNQ1OT1, GDF-15, SREBP1, PXR, TRAF6, MEG3, YAP1	alternative splicing, regulatory T cell, sonic hedgehog, Th17, large B cell, metabolic reprogramming, autophagic flux	Docetaxel, mesenchymal stem cell, cilostazol, barbarum polysaccharide, ginsenoside, everolimus, panax notoginseng, oridonin
3	0.97	mir-204, MPTP, CAS9, JNK, PCAF, mir-181, IL-23, MSCS, STAT6, BOX-1, PKM2, aquaporin, uncoupling, mir-126, mir-29b, MST1, BCL6, ABCA1, CRNDE, IL-1β, AT1R, E2F3, SNHG12, S6K1, GSTM1, CBP, SOX2, PKD1	fecal microbiota, M2 polarization, checkpoint	Budesonide, ketogenic diet, astragalus polysaccharide, triptolide, hesperetin
4	0.96	mir-206, mir-194-5p, mir-23b-3p, mir-212-3p, Mas receptor, AXL, ASK1, Yes associated protein, GRP78, CARM1, Orphan nuclear receptor, BRD4, CX3CL1, SP1, MIAT, MECP2, P2X7, GSTP1, YY1, NFAT, ANGPTL3, SMURF2	—	mediterranean diet, mangiferin, amlodipine, tanshinone
5	0.95	TAK1, SREBP, LOX-1, EGR-1, TLR9, MKK3	—	silymarin, cyclocarya paliurus, ginkgolide, nobiletin, electroacupuncture, artesunate, celecoxib, atractylenolide

## Discussion

### General trends of sirtuins and kidney diseases

In this study, we conducted a holistic evaluation of sirtuin-related research over nearly 2 decades. We found that since 2013, research on sirtuins has exploded in various fields, likely because the crucial role of sirtuins in health and disease has gradually gained the attention of researchers and clinicians. We reviewed the retrieved publications by timeline and identified a few landmark publications, such as Frye, RA, in 1999, which reported five sirtuin (SIRT1-SIRT5) homologs to yeast sir2 and found that these proteins can metabolize NAD (Frye, 1999). NAD^+^ has also received widespread attention as a fuel for sirtuins. In the last 3 years, two of the most cited articles have reported the important role of NAD^+^ homeostasis in disease and health, and elevated NAD^+^ levels have shown beneficial effects in various diseases (Katsyuba et al., 2020; Covarrubias et al., 2021). Cardiovascular diseases and neurodegenerative disorders are the main forces of sirtuin research. A recent highly cited article reported that NAD^+^ supplementation can reverse vascular aging by activating sirt1, and this effect could be further enhanced by hydrogen sulfide supplementation (another dietary restriction mimetic) (Das et al., 2018). Similarly, in another highly cited paper, NAD^+^ supplementation was found to ameliorate neuroinflammation, DNA damage, and synaptic dysfunction in Alzheimer’s disease (Hou et al., 2018). These results illustrate that NAD^+^, an important branch in the study of sirtuins, has received extensive attention, which also provides inspiration for the study of sirtuins in kidney diseases. Notably, in this study, we found that sirt3 is the most widely studied member of sirtuins family after sirt1. Deficiency of sirt3 was shown to result in HIF1α accumulation and PKM2 dimer formation, and subsequently leads to aberrant glycolysis and mesenchymal transformations, ultimately promoting fibrosis in DN (Srivastava et al., 2018). Recently, sirt3 was reported to contribute to the improvement of metabolic reprogramming and endothelial-to-mesenchymal transition (EndMT) in endothelial cells of DN, leading to the amelioration of fibrosis (Srivastava et al., 2021a). Interestingly, deficiency of Fibroblast Growth Factor Receptor 1 (FGFR1) has been reported to mediate the EndMT in DN and aggravate fibrosis (Li et al., 2020). N-acetyl-seryl-aspartyl-lysyl-proline (AcSDKP) restores the expression of sirt3 and ameliorates fibrosis in DN (Srivastava et al., 2020a), an effect that was found to be partially dependent on FGFR1 (Li et al., 2020).

### General trends of sirt1

Sirt1 is the most extensively studied member of the sirtuin family, with more than half of the sirtuin studies focused on sirt1. The United States and China were central participants in sirt1 studies. Harvard Medical School in the United States was a major contributor to sirt1 studies, and Sinclair DA’s team was the representative. They previously reported that RSV acts as a sirt1 activator to mimic calorie restriction, improve the DNA stability of yeast, and prolong its lifespan by 70% (Howitz et al., 2003). Recently, Sinclair DA et al. focused on the significance of NAD^+^ metabolism in disease and health. They found that activating sirt1 or increasing NAD^+^ levels improved the health of patients with cardiovascular and metabolic diseases (Das et al., 2018; Kane and Sinclair, 2018). In addition, they also found that NAD^+^ supplementation maintains telomere length and inhibits DNA damage, thus improving fibrotic disorders and premature aging (Amano et al., 2019). MIT in the United States is another important contributor to sirt1 research, and Guarente L’s team is the leader. They found early that sirt1 can deacetylate and regulate p53 to improve cell aging and apoptosis (Vaziri et al., 2001), and a recent study found that NAD^+^ supplementation rejuvenates stem cells in the aging intestine (Igarashi et al., 2019).

More than one-third of the publications were published in Q1 journals, showing the high academic influence of the research on sirt1, and with Cell, Nature, and Science being the most representative journals. Recently, an article published in Cell reported that the deacetylation of sirt1 produces an important protective effect against non-aging-related brain injury (Shin et al., 2021). Nature has recently reported dynamic changes in miR-34a targeting sirt1 (Baronti et al., 2020). In addition, based on co-cited references and keyword clusters and burst analysis, we found that diabetes, skeletal muscle, cancer, and cognitive deficit are hotspots diseases for the study of sirt1, and NAD^+^, oxidative stress, and deacetylation regulation of p53 are hotspots in the research field of sirt1. In addition, we also found that autophagy, NLRP3 inflammasome, AMPK pathway have received much attention from researchers as emerging research trends in recent years. Both autophagy and sirt1 have been recognized as important players in the aging process, and the connection between them has attracted much interest. Cui et al. (2006) suggested that blockade of autophagy is accompanied by a decrease in sirt1 expression. The most cited articles reported that sirt1, as an important regulator of autophagy, can regulate protein expression *via* deacetylation of autophagy-related genes (*Atg*), such as *Atg5*, *Atg7*, and *Atg8*, while sirt1 deficiency resulted in impaired autophagy activation (Lee et al., 2008). A recent study identified sirt1 as a novel and selective substrate of nuclear autophagy and an important regulator of sirt1 protein homeostasis (Xu et al., 2020), while inhibition of autophagy could promote sirt1-mediated health benefits (Wang et al., 2021). NLRP3 inflammasome are reportedly involved in sirt1. Fu et al. (2013) showed that RSV, as a sirt1 activator, inhibits NLRP3 inflammasome activation, while sirt1 inhibition can significantly enhance the expression of inflammatory factors. The latest findings that the sirt1 agonist SRT1720 reduces NLRP3 inflammasome activation and pyroptosis in an Akt signaling-dependent manner (Han et al., 2020) and that NLRP3 knockout leads to increased NAD^+^ levels and increased sirt1 expression, illustrate that the NLRP3 inflammasome appears to be involved in sirt1-associated-aging.

### Sirt1 and kidney diseases

We found that kidney disease has gained increasing attention in recent years in the study of sirt1. China maintained its leading position both in terms of total publications and total citations, followed by researchers at the United States. Fudan University from China have been working on sirt1, and Hao CM’s team is the main representative among these researchers. They found that sirt1 may serve as a potential pharmacological target for kidney injury (Guan et al., 2017), and that sirt1 activation effectively improves renal fibrosis (Huang et al., 2014), which may be related to the improvement of renal oxidative stress (He et al., 2010). In addition, it is worth mentioning that Japan has made an important contribution to research investigating sirt1 in kidney disease due to the high citation frequency. Koya D from Kanazawa Medical University and Kume S from Shiga University of Medical Science are the main representatives of this group of researchers. Koya D and Kume S were early scholars in the field of kidney disease who focused on sirt1. As early as 2006, they reported that sirt1 deacetylates and regulates p53, and reduces mesangial cell apoptosis (Kume et al., 2006). The most influential article reported that calorie restriction enhances the adaptability of cells in aging kidneys to hypoxia through sirt1-dependent mitochondrial autophagy (Kume et al., 2010). Recently, a review by Koya D et al. discussed the relationship between sirt1 and oxidative stress in nephropathy, emphasizing that sirt1 activation in the kidney may represent a novel therapeutic strategy (Ogura et al., 2021). Furthermore, JASN, PLOS One, and KI are the most influential journals for sirt1 research in kidney disease. A recent publication in JASN reported that short-term treatment with NMN ameliorated the renal injury phenotype and survival of diabetic nephropathy by upregulating SIRT1 expression (Yasuda et al., 2021). Similarly, a recent article published in KI also reported that BF175 may reduce podocyte loss and renal dysfunction in diabetic nephropathy by activating sirt1 (Feng et al., 2021).

Oxidative stress and mitochondrial biogenesis were the main mechanisms involved in kidney disease. “Autophagy,” “acetylation,” and “NLRP3 inflammasome” have received much attention in the field of kidney disease as emerging research trends. It is widely accepted that sirt1 acts as a major driver of autophagy in kidney disease; however, the exact mechanism remains to be explored. As sirt1 is a novel substrate of autophagy, crosstalk between autophagy and sirt1 deserves significant attention in kidney disease (Xu et al., 2020; Wang et al., 2021). In addition, a recent study reported that the AMPK pathway maintains a high basal level of autophagy in podocytes independent of the mTOR pathway (Bork et al., 2020), this appears to provide important evidence for a link between autophagy and sirt1 in kidney disease. Notably, recent studies have shown that acetylation has emerged as an important regulatory mechanism of autophagy, and that acetylation not only regulates autophagy-related proteins but also affects autophagic function through the regulation of histones and transcription factors, indicating that deacetylation of sirt1 plays an important role in kidney disease, especially in autophagy (Xu and Wan, 2022). Crosstalk between sirt1 and the NLRP3 inflammasome plays an important role in kidney disease. Jiang et al. (2021) found that sirt1 knockout aggravated NLRP3 inflammasome activation in a mouse model of aldosterone infusion, and a recent report also found that sirt1 blunted NLRP3 inflammasome activation *via* autophagy to ameliorate IgA nephropathy (Wu et al., 2020). However, the complex connection between sirt1 and the NLRP3 inflammasome in kidney disease is poorly understood.

In addition, some neglected areas of kidney disease deserve attention as potential emerging hotspots in the study of Sirt1, such as immune, metabolic reprogramming and fecal microbiota. Immunity is a major cause of kidney diseases, including membranous nephropathy, lupus nephritis, and IgA nephropathy (Liu et al., 2020). Innate and adaptive immunity are involved in the initiation and maintenance of kidney injury (Dellepiane et al., 2020). Sirt1 has recently been found to act as an important regulator of immune cells and immune responses (Shen et al., 2021), which deserves the attention of researchers studying kidney diseases. In addition, metabolic reprogramming has been an emerging hotspot of kidney disease research in recent years, and changes in fatty acid metabolism and glucose metabolism have been found to be closely related to renal fibrosis (Zhu et al., 2021). Sirt1 plays an important role in energy and metabolic regulation (Ong and Ramasamy, 2018), and therefore, sirt1 may mediate metabolic reprogramming and play an important role in renal diseases. In addition, gut microbes and their derived metabolites were found to promote the progression of kidney disease and the development of severe complications, especially cardiovascular diseases (Mahmoodpoor et al., 2017; Feng et al., 2019). Recent studies have reported that deletion of sirt1 causes alterations in the gut microbiota, and sirt1 may be an important mediator of the interaction between the host and gut microbes (Wellman et al., 2017), which may provide important inspiration for the study of the gut-kidney axis. Notably, some natural ingredients, such as barbarum polysaccharides, Panax notoginseng, oridonin, triptolide, and hesperetin, deserve attention as potential therapeutic agents influencing sirt1 in kidney diseases.

### Implication of sirt1 in diabetic kidney disease research

Cluster and burst analyses showed that DN was a primary focus in sirt1 research. Sirt1 plays an important role in the epigenetic regulations of renal tubules and podocytes in DN. Sirt1 can deacetylate regulated STAT3, p53, FOXO4 and PGC1-α to maintain podocyte function (Nakatani and Inagi, 2016), and activation of sirt1 promotes the expression of PGC1-α in podocytes, thereby ameliorating podocyte injury and proteinuria in DN (Hong et al., 2018). Similarly, sirt1 could also deacetylate regulated Beclin1 and p53 to improve tubular autophagy (Deng et al., 2021; Sun et al., 2021) and attenuate high glucose induced tubular injury and apoptosis (Wang et al., 2016). Interestingly, renal tubular sirt1 was found to regulate the expression of Claudin-1 to mediate crosstalk with podocytes to ameliorate proteinuria in DN (Hasegawa et al., 2013). In addition, recent studies have found that glucocorticoid receptor (GR) plays an important role in DN. Deficiency of GR of endothelial accelerates renal fibrosis in DN mice (Srivastava et al., 2021c), and the same results were found in DN mice with podocyte GR loss (Srivastava et al., 2021b). Importantly, sirt1 was found to be a transcriptional enhancer of GR and is independent of its deacetylase activity role (Suzuki et al., 2018). Interestingly, some emerging drugs also hold promise for improving DN through sirt1, such as glycolysis inhibitors (Lv et al., 2018), DPP-4 inhibitors (linagliptin) (Elbaz et al., 2018), JAK/STA3 inhibitors (Wang et al., 2018), mineralocorticoid receptor agonists (Moore et al., 2012) and N-acetyl-seryl-aspartyl-lysyl-proline (Srivastava et al., 2020a).

### Pharmacological prospect of sirt1 in kidney disease

**FIGURE 8:**
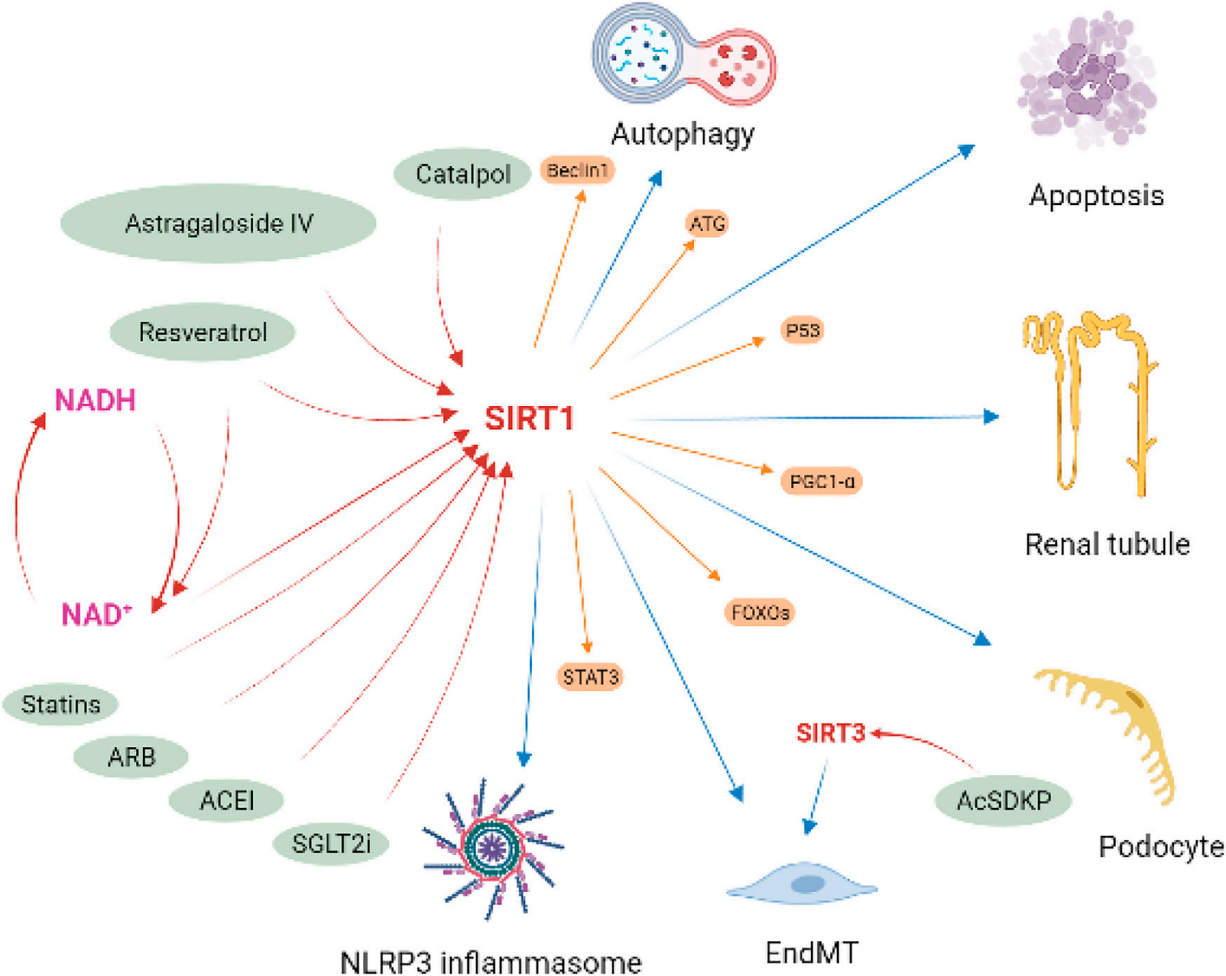
Implication of sirt1 in diabetic kidney disease. Sirt1 attenuated podocyte and tubule injury and apoptosis, improve endothelial-to-mesenchymal transition (EndMT), and regulated autophagy and NLRP3 inflammasome activation in DKD by deacetylating regulatory ATG, Beclin1, STAT3, p53, FOXOs and PGC1-α. Several drugs available in kidney disease have been shown to be partially contribute to the activation of the sirt1, including SGLT2i, ACEI, ARB, and statins. Some natural compounds have also been found to have the same effect, such as resveratrol, catalpol and astragaloside IV.

In summary, sirt1 plays an important role in the regulation of inflammation, autophagy, oxidative stress, and mitochondrial homeostasis in kidney disease, especially in DN. *In vivo* and *in vitro* evidence shows that Sirt1 can serve as an important potential pharmacological target in kidney disease (Figure 8). Several drugs available in kidney disease have also been shown to be partially contribute to the activation of the sirt1, such as sodium glucose co-transporter two inhibitors (Packer, 2020), angiotensin-converting enzyme inhibitor (enalapril) (Veitch et al., 2021), angiotensin Ⅱ receptor blocker (olmesartan) (Gu et al., 2016), and statins (Khayatan et al., 2022). In addition, some small molecule compounds, especially some natural compounds, targeting sirt1 may also serve as potentially promising candidates for the treatment of kidney diseases, like RSV, Catalpol and Astragaloside IV.

### Limitation

Compared with the traditional review, bibliometrics is more tend to objective sort out a research field as a whole, which is suitable for the beginning of a research, to find the mainstream direction and emerging hot spots, so as to make more meaningful research. This study suggests our sirt1 as highly promising in the study of kidney diseases, especially in DN, and the relationship between Sirt1 and immune, metabolic reprogramming and fecal microbiota in kidney diseases deserves significant attention. However, this study has some limitations that need to be considered. First, we developed a search formula in as much detail as possible; however, that is difficult to avoid some studies were not included in our analysis. Second, we selected as many landmark articles as possible; however, it is possible that some important publications and research directions were missed due to differences in the evaluation indicators on which we focused. Finally, we combined various bibliometric analysis methods in order to obtain more useful and instructive results, but due to the limitations of the algorithm, some newer views may not be presented.

## Conclusion

Based on bibliometric analysis, we found that publications regarding sirt1 have increased dramatically in the last 2 decades, especially in the last 5 years, in which studies on kidney diseases have gained increasing attention. China and the United States are major contributors to sirt1 studies, and Japanese scholars have also made important contributions to studies about sirt1 in kidney disease. NAD^+^, oxidative stress, and p53 are the main focus of scholars studying sirt1. Autophagy and NLRP3 inflammasome are emerging research trends that have gradually attracted the interest of researchers, especially regarding kidney diseases. In addition, some neglected potential research topics deserve further attention and in-depth studies.

## Data Availability

The original contributions presented in the study are included in the article/Supplementary Material, further inquiries can be directed to the corresponding author.
