# Supplementary material for: Comprehensive bibliometric analysis of sirtuins: Focus on sirt1 and kidney disease
Source: Front Pharmacol. 2022 Aug 16;13:966786. doi: 10.3389/fphar.2022.966786 (PMC9424666; doi:10.3389/fphar.2022.966786)
Supplement: Supplementary file 1 [file DataSheet1.docx]

**Supplementary Materials**

Table S1. Retrieval of classified items

| Item | Retrieval formula | Duration | Number of publications |
| --- | --- | --- | --- |
| Sirt1 | TS=(“SIRT1” OR “sirtuin1”) | 1999-2022 | 11265 |
| Sirt2 | TS=(“SIRT2” OR “sirtuin2”) | 1999-2022 | 1061 |
| Sirt3 | TS=(“SIRT3” OR “sirtuin3”) | 2000-2022 | 2049 |
| Sirt4 | TS=(“SIRT4” OR “sirtuin4”) | 1999-2022 | 289 |
| Sirt5 | TS=(“SIRT5” OR “sirtuin5”) | 1999-2022 | 390 |
| Sirt6 | TS=(“SIRT6” OR “sirtuin6”) | 2000-2022 | 1039 |
| Sirt7 | TS=(“SIRT7” OR “sirtuin7”) | 2000-2022 | 363 |
| Sirt1 in kidney disease | TS=(“SIRT1” OR “sirtuin1”) AND TS=("kidney" OR "renal" OR "nephropathy") | 2006-2022 | 744 |

Table S2. The top 10 highest productive countries for the study of sirt1 in kidney disease

| Rank | Country | Number of publications | Total times cited | Average citation frequency | H-index |
| --- | --- | --- | --- | --- | --- |
| 1 | China | 417(56.05%) | 9099 | 21.82 | 49 |
| 2 | United States | 147(19.76%) | 6932 | 47.16 | 48 |
| 3 | Japan | 54(7.26%) | 3223 | 59.69 | 26 |
| 4 | South Korea | 51(6.86%) | 1649 | 32.33 | 23 |
| 5 | Iran | 23(3.09%) | 158 | 6.87 | 6 |
| 6 | Egypt | 19(2.55%) | 147 | 7.74 | 7 |
| 7 | Italy | 18(2.42%) | 1006 | 55.89 | 11 |
| 8 | Germany | 16(2.15%) | 547 | 34.19 | 11 |
| 9 | India | 14(1.88%) | 208 | 14.86 | 8 |
| 10 | Poland | 14(1.88%) | 248 | 17.71 | 8 |

Table S3. The top five most cited authors for the study of sirt1 in kidney disease.

| Rank | Author | Institution | Country | Number of publications | Total times cited | Average citation frequency | H-index |
| --- | --- | --- | --- | --- | --- | --- | --- |
| 1 | Koya D | Kanazawa Medical University | Japan | 16 | 1799 | 112.44 | 14 |
| 2 | Kume S | Kanazawa Medical University | Japan | 11 | 1446 | 131.45 | 10 |
| 3 | Uzu T | Shiga University of Medical Science | Japan | 5 | 855 | 171.00 | 5 |
| 4 | Kashiwagi A | Shiga University of Medical Science | Japan | 4 | 803 | 200.75 | 4 |
| 5 | Kitada M | Kanazawa Medical University | Japan | 9 | 774 | 86.00 | 7 |

Table S4. Top ten journals with the largest number of publications and the most cited related to sirt1 in kidney disease.

| Rank | Journal | Number of publications | Total times cited | Average citation frequency | Journal(cited) | Number of publications | Total times cited | Average citation frequency |
| --- | --- | --- | --- | --- | --- | --- | --- | --- |
| 1 | Frontiers in Pharmacology | 18 | 165 | 9.17 | Plos One | 18 | 1305 | 72.50 |
| 2 | Plos One | 18 | 1305 | 72.50 | Journal of Clinical Investigation | 4 | 1096 | 274.00 |
| 3 | Scientific Reports | 16 | 389 | 24.31 | Journal of the American Society of Nephrology | 12 | 874 | 72.83 |
| 4 | Biochemical And Biophysical Research Communications | 14 | 443 | 31.64 | Aging Cell | 8 | 810 | 101.25 |
| 5 | Frontiers in Physiology | 12 | 136 | 11.33 | Kidney International | 10 | 611 | 61.10 |
| 6 | International Journal of Molecular Sciences | 12 | 150 | 12.50 | Journal of Biological Chemistry | 4 | 525 | 131.25 |
| 7 | Journal of the American Society of Nephrology | 12 | 874 | 72.83 | Diabetes | 3 | 493 | 164.33 |
| 8 | American Journal of Physiology-Renal Physiology | 10 | 373 | 37.30 | Free Radical Biology and Medicine | 7 | 446 | 63.71 |
| 9 | Kidney International | 10 | 611 | 61.10 | Biochemical And Biophysical Research Communications | 14 | 443 | 31.64 |
| 10 | Oxidative Medicine and Cellular Longevity | 10 | 316 | 31.60 | Scientific Reports | 16 | 389 | 24.31 |
